# Parents’ Responses and Reactions to the National Childhood Measurement Programme in a Contemporary Sample of British Children: A Mixed‐Method Study

**DOI:** 10.1155/jobe/1001038

**Published:** 2025-12-08

**Authors:** Florence Sheen, Alice Kininmonth, Zeynep Nas, Alison Fildes, Clare Llewellyn

**Affiliations:** ^1^ School of Sport, Exercise and Health Sciences, National Centre for Sport and Exercise Medicine, Loughborough University, Loughborough, LE11 3TU, UK, lboro.ac.uk; ^2^ School of Food Science and Nutrition, University of Leeds, West Yorkshire, LS2 9JT, UK, leeds.ac.uk; ^3^ Department of Behavioural Science and Health, Institute of Epidemiology & Health Care, University College London, 1-19 Torrington Place, London, WC1E 7HB, UK, ucl.ac.uk; ^4^ School of Psychology, University of Leeds, West Yorkshire, LS2 9JT, UK, leeds.ac.uk

**Keywords:** child health, childhood obesity, child weight, national child measurement programme, parent perspectives, weight measurement, weight perceptions

## Abstract

**Background:**

Parents’ support for the National Childhood Measurement Programme (NCMP, England) is typically mixed, with qualitative data from small samples of self‐selecting parents highlighting different responses depending on whether their child is classified as a ‘healthy’ or alternative weight.

**Objective:**

Using data from the Gemini cohort study, we explored parents’ emotional and behavioural responses to feedback on their children’s weight status.

**Methods:**

We performed secondary data analysis on data collected when children were 12–13 years old (2019–2021). Their parents completed a questionnaire online, which included (optional) questions about the NCMP. Parents indicated the weight status assigned to their child by the NCMP and answered questions about the weight feedback, including an open‐ended question about their emotional responses.

**Results:**

There were 567 complete responses to the NCMP questions (55.8% of parents who completed the questionnaire). The majority of children were categorised as ‘healthy weight’ (*n* = 440, 77.6%). Among parents whose children were categorised as an alternative weight status (*n* = 101), 29.7%, 54.5% and 45.0% of those with children categorised as underweight, overweight or very overweight, respectively, reported that they took action following the NCMP feedback. Qualitative results highlighted emotional responses and inferences made about children’s well‐being based on the feedback received; parents usually reacted positively (happy, inferred their child was healthy) if their child was categorised as a healthy weight, and negatively (worried, inferred their child was not healthy) if their child was categorised as an alternative weight status. There was also some distrust of the feedback and the NCMP itself.

**Conclusion:**

The negative reactions of many parents to NCMP feedback, and the programme itself, highlight the need to involve parents and caregivers in the delivery of the NCMP and framing of feedback. Parent and caregiver input is vital to understand how best to communicate information about children’s weight to parents, signpost them to support and what support pathway should be implemented to increase parent uptake.

## 1. Background

The prevalence of obesity in the United Kingdom remains high, particularly in children and adolescents; in 2023–2024, 22% and 35.9% of children aged 4–5 and 10–11, respectively, were living with overweight or obesity in England [1]. Having obesity as a child is associated with increased likelihood of obesity in adulthood [2, 3], premature morbidity and mortality [4], as well as increased risk of comorbidities, such as some cancers [5], cardiovascular complications [6], and depression [7]. Intervening to prevent and treat overweight and obesity in childhood has the potential to improve both current and future health.

The English National Childhood Measurement Programme (NCMP) measures the heights and weights of children in their first (Reception, 4–5 years) and last (Year 6, 10–11 years) years of primary school, and includes approximately 95% of eligible children (∼1 million children annually); Wales, Scotland and Northern Ireland have similar programmes. The chief aim of the NCMP is to monitor the prevalence of overweight and obesity in children. While NCMP measurement is mandatory for Local Authorities (LAs) to implement across England, they can also opt to communicate the children’s height and weight, or weight status, to parents and caregivers, and most LAs (86%) choose to do this [8]. Parents typically receive their child(ren)’s feedback in a letter (designed by DHSC with input from researchers), which includes their child’s height and weight measurements or weight status, and information about supporting children to be physically active, eat healthily and how to talk to children about their weight; some LAs opt to telephone parents to discuss results directly. Most LAs (80%) refer families to weight management services, where appropriate and if available [8].

Research exploring parental responses to the NCMP has typically shown them to be negative, with some suggesting that the NCMP contributes to weight stigma [9, 10]. A recent meta‐ethnography investigating the NCMP (the experience of being measured and receiving results) indicated that children categorised as overweight or obese perceive the NCMP to be an emotionally significant event that changes their relationships with their body, food and their peers [9]. A pre‐post survey of parents of children aged 4–5 and 10–11 years further highlighted this disparity in impact for parents and caregivers, with substantially more parents of children categorised as outside of the ‘healthy’ weight range reporting feeling upset, guilty or angry, compared to parents of children classified as ‘healthy’ weight [11]. For parents whose children were classified as overweight or obese, general awareness of childhood overweight as a health issue increased and more than a third sought further information regarding their child’s weight. In addition, there was a significant increase in the proportion of parents who correctly recognised childhood overweight or obesity (increases of 11.1% and 23.5% respectively). However, reported changes in lifestyle behaviours following NCMP feedback were inconsistent (between −9.9% and 12.6% reporting change to any of the listed lifestyle behaviours), with the only significant change being increased physical activity among children already living with obesity [11].

In a focus group with a purposive sample of parents who recently received NCMP feedback, parents whose child had been classified as overweight or obese often rejected the feedback, because they felt it lacked credibility [12]. Similarity, in semi‐structured interviews with parents of children aged 4–5 and 10–11 years who had been categorised as overweight or obese by the NCMP, feedback was perceived as unreliable because it did not consider the individual child’s lifestyle; they also considered ‘health and happiness as more important than weight’ [13]. Researchers [14, 15] and LAs (unpublished data) have also been concerned about the current growth reference data used to classify the weight status of children in the NCMP (UK90), because it does not account for ethnicity, which may compromise the reliability of weight status classification for some children [14].

Current research exploring parents’ responses and reactions to the NCMP has involved either quantitative questionnaire approaches [11] or qualitative group approaches with smaller numbers of self‐selecting parents, who may have stronger opinions about the NCMP [12, 13]. To extend this previous research, the current study employed a mixed‐methods approach using quantitative and qualitative data collected in the Gemini study [16], a large population‐based British birth cohort of twins, to explore parents’ perspectives. To our knowledge, this is the first mixed‐methods study of the NCMP in a large sample. Specifically, we examined parents’ emotional and behavioural responses to feedback on their child(ren)’s weight status from the NCMP, which were collected when the twins were 12–13 years old. This research aimed to address the question: ‘How do parents respond to being told that their child has been categorised as a certain weight status?’

## 2. Methods

### 2.1. Participants

Data collection took place between 2019 and 2021, when the twins were 12–13 years old (12.70 ± 0.44). There were 508 parents of twin children from the Gemini cohort who completed questionnaires at this time point (*N* = 1016 children), with responses to the NCMP questions for 567 children.

### 2.2. Procedure

Detailed information regarding Gemini data collection procedures and measures have been published elsewhere [16]. Participants completed the questionnaires online, and the NCMP questions were an optional (i.e. not ‘forced response’) set of questions, alongside a battery of other measures related to children’s health and development. We followed the Standards for Reporting Qualitative Research (SRQR) (see Supporting File 1).

### 2.3. Measures

Parents completed the questionnaire for each of their twins separately. Baseline data (the first surveys completed when the twins were approximately 9 months old) assessed seven indicators of socioeconomic position (SEP), used to create a composite score, spanning individual (i.e. maternal educational qualifications, current occupation), household (i.e. annual household income, number of bedrooms and cars, home ownership status) and neighbourhood‐level (postcode for calculating index of multiple deprivation) factors. Higher scores on the composite score represent higher SEP [17].

A series of questions were included to capture parents’ experiences of the NCMP. Parents were asked to indicate the weight status their children had been assigned by the NCMP from categories of ‘underweight,’ ‘healthy weight,’ ‘overweight’ and ‘very overweight.’ Parents were able to select ‘don’t remember’ or leave this question blank. Parents indicated whether they agreed with the NCMP feedback (yes/no), and what they believed their child’s weight status to have been at that time using the same weight categories. Participants were asked ‘How did the feedback regarding [Twin’s name] make you feel?’ with an open text response box. Parents indicated whether they had shared the NCMP feedback with their child (yes/no) and whether they took any actions following the feedback (yes/no). If parents selected ‘yes’ for the latter question, they were asked to select all the actions they had taken from the following list: ‘contacted a health professional (e.g. GP or school nurse),’ ‘accessed online resources about healthy lifestyles,’ ‘encouraged my child to eat more healthily,’ ‘encouraged my child to be more active’ and ‘enrolled my child in a healthy weight programme.’

### 2.4. Quantitative Analyses

Descriptive statistics were used to explore the demographics of the sample, parental agreement with weight status assignment, whether parents shared the weight status with their child(ren), whether parents opted to take action and the reported actions taken. Chi‐squared tests explored differences in agreement and action taken by weight status category and explored whether there was a relationship between agreement with the NCMP feedback and taking action.

### 2.5. Qualitative Analyses

We used reflexive thematic analysis [18, 19] to explore the open‐ended responses to the question ‘How did the feedback regarding [twin’s name] make you feel?’

 
*Step 1 (Familiarisation):* Give the amount of qualitative data (*n* = 451), Excel was used to code the data for ease of appraisal across the whole sample. F.S. familiarised herself with the data, keeping reflective notes on initial ideas.
 
*Step 2 (Generating initial codes):* F.S. generated initial codes, inductively coding each statement made about each child.
 
*Step 3 (Searching for themes):* Candidate themes were first explored across the whole dataset by F.S., who used thematic maps to support the grouping of codes into broader themes. These were presented to the wider research team (A.K., Z.N., C.L. and A.F.) for discussion before Step 4 commenced.
 
*Step 4 (Reviewing themes):* To review these themes, F.S. separated the data into three groups; parents whose child(ren) had been classified as ‘underweight’ (*n* = 35), ‘healthy weight’ (*n* = 358) or ‘overweight’ (*n* = 58) (note, because of the small number of children classified as ‘very overweight’ in the qualitative sample (*n* = 18), the categories ‘overweight’ and ‘very overweight’ were consolidated into one group named ‘overweight’). Here, she addressed the inductive coding for each group and reappraised the candidate themes to capture relationships between the weight status assigned to the child and parents’ responses. A.K. and Z.N. then second‐coded 70% of the data and discussed with F.S. to ensure consistency and explore interpretations. Positionality statements were completed by F.S., A.K. and Z.N. prior to their engagement with the data (see Supporting Information File 2).
 
*Step 5 (Defining and naming themes):* The author team (F.S., A.K., Z.N., A.F. and C.L.) discussed the cultivated themes and agreed on theme names. FS finalised the themes and wrote the initial draft of the results section, which was reviewed by the wider author team (A.K., Z.N., C.L. and A.F.).

## 3. Results

### 3.1. Participants

Of the responses at this wave of data collection (*N* = 1016), there were 567 complete responses to the NCMP questions (i.e. the question about their child’s weight status had not been left blank, 55.8% of parents had completed the longer Gemini questionnaire at this time point). Demographic information for this sample is reported in Table 1 (see Supporting Information File 3 for twin‐specific demographic information, e.g. zygosity). There were roughly equal numbers of males and females (51.5% female), with a mean age of 12.7 years (±0.44). The majority of children were of White British ethnicity (*n* = 493, 86.9%), and most parent responders were mothers (98.9%), with only six fathers. The majority of children were categorised as ‘healthy weight’ (77.6%), with 17.8% categorised as an alternative weight status (‘underweight’ (*n* = 37), ‘overweight’ (*n* = 44) or ‘very overweight’ (*n* = 20)). A small proportion of parents could not remember their child’s assigned weight status (4.6%) (see Table 1). Of the twin pairs for whom a weight status was given for both twins (i.e. not ‘don’t remember’ or data missing for one or both twins, *n* = 552) 75.7% were congruent for weight status (see Supporting Information File 3, Table S3).

**Table 1:** Children’s demographics.

	Mean (±SD)	
Age (years)	12.70 (±0.44)	
Socioeconomic position (SEP)^1^	4.78 (±1.10)	

	**Frequency**	**%**

Gender		
Female	292	51.5
Male	275	48.5
Ethnicity		
White British	493	86.9
Other white	6	1.1
Asian Indian	6	1.1
Asian Bangladeshi	2	0.4
Mixed ethnicity	60	10.6
NCMP weight status^2^		
‘Underweight’	37	6.5
‘Healthy weight’	440	77.6
‘Overweight’	44	7.8
‘Very overweight’	20	3.5
‘Don’t remember’	26	4.6

^1^Socioeconomic position (SEP) composite score from baseline data.

^2^Categories refer to those used in the NCMP letter, where children are assigned to a weight status of either ‘underweight’, ‘healthy weight’, ‘overweight’ or ‘very overweight’.

### 3.2. Parents’ Responses to NCMP Feedback

The majority of parents whose children were categorised as ‘healthy weight’ (97.3%) or ‘underweight’ (78.4%) agreed with the assigned category. For parents whose children were categorised as ‘overweight’ or ‘very overweight,’ the proportion of parents disagreeing was higher (40.9% and 50.0%, respectively) (see Figure 1 and Supporting Information File 3).

Figure 1Parental agreement (a), whether they shared the weight feedback with their child (b) and action taken following the feedback (c) split by assigned weight category.

**Figure:**
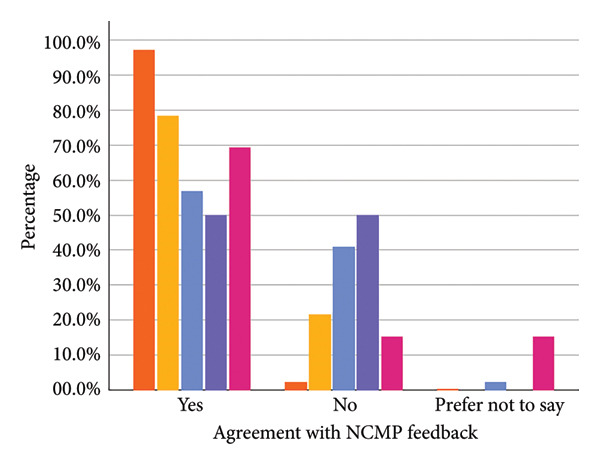
(a)

**Figure:**
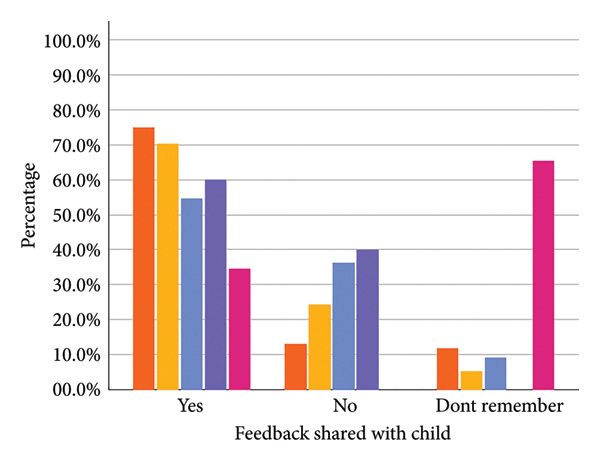
(b)

**Figure:**
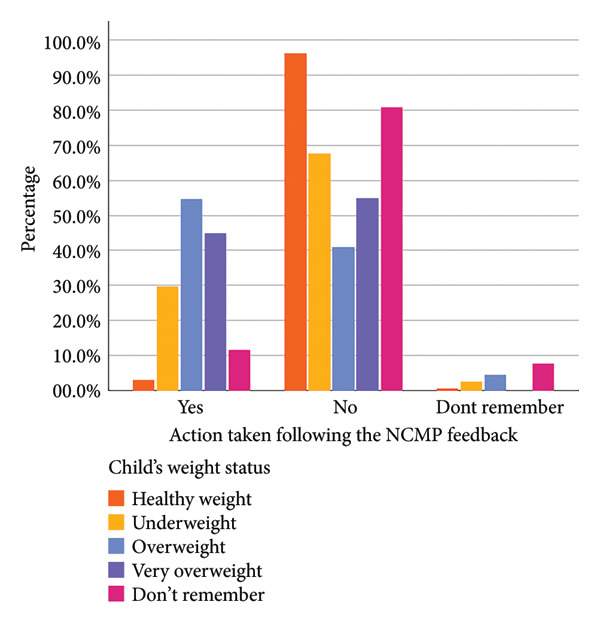
(c)

A substantial proportion of parents whose children were categorised as an alternative weight status (*n* = 101) reported that they took action following the NCMP feedback: 29.7% for underweight, 54.5% for overweight and 45.0% for very overweight (see Supporting Information File 3, Table S4). The most common actions were to encourage the child to eat more healthily or to be more active; only one parent reported enrolling their child (classified as ‘very overweight’) in a ‘healthy weight’ programme (see Table 2). For parents whose children were categorised as underweight, the most popular course of action was to contact a healthcare professional.

**Table 2:** Parents who took action split by weight category assigned by NCMP (Values in brackets are %).

	Contacted a health professional (e.g. GP or school nurse)	Accessed online resources about healthy lifestyles	Encouraged my child to eat more healthily	Encouraged my child to be more active	Enrolled my child in a ‘healthy weight’ programme
‘Underweight’ (*n* = 11)	8	2	2	2	0
‘Healthy weight’ (*n* = 14)	2	2	9	5	0
‘Overweight’ (*n* = 24)	1	1	22	17	0
‘Very overweight’ (*n* = 9)	4	1	6	6	1
‘Don’t remember’ (*n* = 3)	0	0	3	3	0
Total^1^	15 (2.6%)	6 (1.1%)	42 (7.4%)	33 (5.8%)	1 (0.2%)

*Note:* Table 2 contains all parents who answered ‘yes’ to whether they took action following the NCMP feedback and selected at least one specific action; parents could select multiple actions from the list of five specified actions.

^1^Values in brackets are percentage of the sample (*N* = 567) who selected doing this behaviour.

Chi‐squared tests indicated that parents whose child was categorised as an alternative weight status were less likely to agree with the NCMP feedback (*χ* ^2^ (6) = 137.06, *p* < 0.001) and were more likely to report taking action (*χ* ^2^ (6) = 161.61, *p* < 0.001). In addition, parents typically did not report taking action if they agreed with the feedback, *χ* ^2^ (4) = 51.53, *p* < 0.001 (see Supporting Information File 3, Table S4).

### 3.3. Thematic Analysis

Of the 567 children who had NCMP measurements reported, there were 451 text responses to the question ‘How did the feedback regarding [twin’s name] make you feel?’ (parents of 35 children categorised as ‘underweight,’ 58 as ‘overweight’ and 358 children as ‘healthy weight’). Our thematic analysis cultivated 4 themes and 12 subthemes (see Table 3).

**Table 3:** Themes and illustrative quotes.

Overarching theme	Theme	Sub‐theme	Quote
—	Healthiness perceived as an achievement parents should fulfil	Positive emotions and perceived success	*‘Happy and proud’* (P38551, ‘Healthy Weight’). ‘*Pleased and reassured’* (P28092, ‘Healthy Weight’). *‘Good – was glad they were healthy’* (P14611, ‘Healthy Weight’). *‘…glad he isn’t overweight.’* (P40892, ‘Healthy Weight’). *‘Happy that she was a suitable weight according to professionals’* (P23521, ‘Healthy Weight’).
		Negative emotions and concerns for child health	*‘Rubbish, demoralised and awful as a mum’* (P26571, ‘Underweight’). *‘Concerned and worried’* (P10362, ‘Overweight’). *‘That we need to get our twins to eat healthy and exercise more’ (*P12771, ‘Very Overweight’). *‘…suggested she went to a health class for 6 weeks’* (27932, ‘Very Overweight’). *‘I have consulted a doctor…’* (P32351, ‘Underweight’).

Framing the feedback	Justification	Comparisons	*‘I can see quite clearly that [Child Name] is bigger than his brother’* (P37651, ‘Healthy Weight’). *‘[Child Name] like myself is heavy - and always has been’* (P12361, ‘Overweight’). *‘…do sometimes (compared to their peers) look very slim’* (P19351, ‘Healthy Weight’).
		They are healthy	*‘Both children are large framed/large stature. Both follow a healthy diet and play sports’* (P14231, ‘Overweight’). *‘[Child Name] is very healthy, fit and strong and eats a very good diet so never concerned’* (P17201, ‘Overweight’). *‘not concerned, as I know they eat sensibly and in line with how hungry they are and their activity levels and their body type’* (P34361, ‘Underweight’).
		There are other explanations	*‘Concerned but she is a full time wheelchair user’* (P10712, ‘Very Overweight’).
	Distrusting the assigned weight category	Questioning the feedback	*‘It was good feedback but I expected the twins to be both classed as underweight due to their size’* (P19931, ‘Healthy Weight’). *‘…he seems a very slim child…so I was surprised how near he was to ‘overweight’* (P33621, ‘Healthy Weight’).
		Measurement details	*‘he was very low %ile and borderline but it said healthy’* (P21012, ‘Healthy Weight’). *‘as he was only just in the overweight category I was not overly worried…’* (P25652, ‘Overweight’). *‘It was as expected but I was a little concerned that she was right at the bottom end of “normal”’* (P26652, ‘Healthy Weight’).
		Measurement is incorrect	*‘Not believing it’* (P26942, ‘Overweight’). *‘I don’t think it was an accurate measurement as she had been weighed as lighter than [Twin Name], which she has never been…’* (P43052, ‘Underweight’).
	The programme is the problem	Negative impacts on (their) children	*‘…I was worried about the effect it would have on him as he is self-conscious about his weight – he compares himself to his brother.’* (P25652, ‘Overweight’). *‘It really upset [Child Name] & she still talks about it now. I think it was damaging, in the sense that they were branded overweight & now constantly obsess that they are “fat”’* (P13411, ‘Overweight’). *‘…impressionable [] + potentially damaging to self-esteem’* (P20801, ‘Healthy Weight’). *‘…Unfair to make them worry about what they look like at 11 years old’* (P43012, ‘Overweight’).
		Not useful	*‘It didn’t tell me anything helpful’* (P26681, ‘Healthy Weight’). *‘I knew already so it didn’t come as a surprise’* (P43341, ‘Underweight’).
		Lack of underweight support	*‘…felt they should have made suggestions or recommendations’* (P10862, ‘Underweight’). *“…had hoped for some guidance and tips on how to improve his weight”* (P18911, ‘Underweight’). *‘…The system is only set up to flag overweight children and we were very unimpressed…’* (P32881, ‘Underweight’).
		Disagree with weight measurement approach	*‘Too much focus on weight but not looking at full facts, i.e., height, shoe, size, []* etc.’ (P11931, ‘Very Overweight’). *‘I think you have to balance what the scales are telling you along with the body shape and dress size to truly try identify if your child unhealthily underweight or overweight’* (P12361, ‘Overweight’). *‘I don’t feel this was necessary to do at school’* (P28281, ‘Healthy Weight’).

*Note:* Table 3 contains each theme, subtheme and illustrative quotes.

#### 3.3.1. Theme 1: Healthiness Perceived as an Achievement Parents Should Fulfil

This theme encompasses the subthemes ‘Positive emotions and perceived success related to child weight’ and ‘Negative emotions and concerns for child health’. Parents’ responses tended to relate to whether their child was categorised as ‘healthy weight’ or an alternative weight status. While being categorised as ‘healthy weight’ tended to bring out positive emotions, positive inferences about their child’s health and no call to action, being categorised as an alternative weight status resulted in more negative emotions, inferring a problem or a call to (alluded) action.

The most common response to the open‐ended question was to give a word describing the emotion felt, for instance ‘*happy’*, ‘*reassured’*, ‘*sad’* or ‘*annoyed’*. Positive emotions were typically elicited by a ‘healthy weight’ categorisation, whereas only negative emotions were associated with any alternative weight category. Positive emotions centred around feeling happy, proud or relieved and would often relate to inferences made from the feedback received. In particular, parents often stated they were happy because of what they inferred from the ‘healthy weight’ category, either that their child’s weight is healthy, ‘correct’ or ‘consistent’; ‘*was happy to know that they are at an acceptable weight for their age’* (P38701, ‘Healthy weight’); or that they were doing ‘well’ or ‘growing well’. Parents highlighted being happy that their child was not classified as ‘underweight’ or ‘overweight,’ with some indicating that they had expected them to be, ‘*Relieved as in a home check beforehand he came out overweight’* (P36992, ‘Healthy weight’). It is also worth noting that explicit statements of parental responsibility, although few in our sample, were typically presented as inferred from positive or negative feedback, ‘*Bad as this means that I am not doing my job properly as a Mum’* (P43421, ‘Overweight’). Concern was sometimes voiced if the twins were discordant for weight status, which led to confusion (e.g. if one twin was categorised as a ‘heathy weight’ and the other as an alternative weight), or from not believing the assigned weight status (e.g. assigned ‘healthy weight’ but expecting them to be categorised as ‘underweight’).

All emotions reported by parents whose child was categorised as underweight or overweight were negative. Negative emotions included ‘anger’, ‘disappointment’ and ‘concern’, with ‘anger’ and ‘disappointment’ directed at the feedback itself, ‘*angry… to make them worry about what they look like at 11 years old’* (P43012, ‘Overweight’), whereas concern was directed toward the child, ‘*I do worry about [Child Name]’s size as he feels small’*. (P36952, ‘Underweight’). For parents of children categorised as overweight, emotional responses were typically ‘anger’, ‘sadness’ or ‘concern’, whereas for individuals categorised as underweight, ‘worry’ or ‘concern’ (but not anger) were typical. Parents whose child was categorised as an alternative weight status were usually concerned because they inferred that there was a problem with their child’s current lifestyle or behaviours that was impacting their health, ‘*A little uneasy about second born as I felt she is a good eater and likes to be active*.’ (P36952, ‘Underweight’). For example, that they were ‘eating too much’ or ‘not doing enough physical activity,’ ‘*[Twin Name] doesn’t do much exercise at all’* (P35991, ‘Overweight’). Some parents felt they needed to act, including seeking advice or support with behaviour change relating to diet or activity. Some parents mentioned changing behaviour as a result of the feedback, for example changing diet or suggesting health classes, ‘*…cut back on sugary food and got him to find an exercise he enjoyed’* (P27851, ‘Overweight’). A small number indicated that they already weigh their children at home (however, Gemini families are invited to submit anthropometric data regularly for the study, so may be more likely to be weighing their children at home). Some parents alluded to seeking advice or support from a healthcare practitioner. Among the parents who provided qualitative responses, the subtheme of ‘take action’ was only present for parents whose child had been categorised as an alternative weight status (although in the larger quantitative sample, a few parents whose child was classified as healthy weight reported taking action). In spite of seeking advice or changing lifestyle behaviours being frequently suggested in NCMP letters to parents of children categorised to a weight status other than healthy, only a small number of our sample mentioned making changes (see also quantitative results).

### 3.4. Overarching Theme: Framing the Feedback

This overarching theme encompasses the themes of ‘Justification,’ ‘Distrusting the assigned weight category’ and ‘The programme is the problem.’

#### 3.4.1. Theme 2: Justification

This theme has subthemes of ‘They are healthy,’ ‘There are other explanations’ and ‘Comparisons.’ Parents typically contextualise the NCMP feedback in relation to the child’s eating behaviour, physical activity levels, physical build or in terms of comparison with others. Although this framing was sometimes seen when a child was categorised as ‘healthy weight’ to give more context, this framing occurred more frequently among parents with a child categorised as alternative weight status.

Parents often cited that their child was healthy; for instance, they follow a healthy lifestyle, are active or have a balanced diet as justification for their dismissal or low concern over an alternative weight status, ‘…*not concerned, as I know they eat sensibly and in line with how hungry they are…’* (P34361, ‘Underweight’). Parents sometimes gave other explanations for the weight status that were not health‐ or lifestyle‐related, the most common of which was highlighting their child’s physical build (e.g. tall, fit, strong), ‘*[Child Name] is Very healthy, fit and strong and eats a very good diet so never concerned’* (P17201, ‘Overweight’). For a small number, additional health concerns were cited to give context to weight feedback. Parents often made comparisons to explain their child’s weight status. This included physical comparisons to the child’s twin or to others, such as one of their parents or children in their class at school, ‘*his father was very much the same at his age so this did not concern us too much’*. (P37281, ‘Underweight’). Usually, comparisons were used to frame the weight category, either to justify their dismissal of the feedback or to highlight why they were unsurprised by it. This also included comparing the child to their own ‘normal,’ highlighting their child has always been this weight status.

#### 3.4.2. Theme 3: Distrusting the Assigned Weight Category

Some parents whose children had been categorised as ‘healthy weight’ questioned the feedback, ‘*It was good feedback but I expected the twins to be both classed as underweight due to their size’* (P19931, ‘Healthy weight’). Some parents remained concerned about their child’s weight even when classed as a ‘healthy weight,’ often because their child’s appearance did not match expectations, for example, expecting a ‘*very slim child’* (P33621, ‘Healthy Weight’) to be at the lower end of ‘healthy weight’ or to have been categorised as having underweight.

Some parents would give further details regarding their child’s measurement, e.g., that they were on the borderline of a category, or the high or low end of a category. Sometimes this was used to explain their concern or lack of concern over the feedback, for instance that the child was on a weight status boundary, ‘*as he was only just in the overweight category I was not overly worried…’*) (P25652, ‘Overweight’). Although rare, some parents whose children were categorised as overweight reported disagreeing with the feedback or not believing it. Some referred to believing the measurement itself was incorrect, while others did not state a reason for their disbelief.

#### 3.4.3. Theme 4: The Programme Is the Problem

Some parents used this space to give opinions on measuring height and weight in school settings and communicating this weight status to parents and, by extension, to the child. Concerns about negative impacts on children were often highlighted, with some parents observing negative impacts on their own child(ren) following participation in the NCMP, ‘*It really upset [Child Name] & she still talks about it now. I think it was damaging*…’ (P13411, ‘Overweight’). For instance, parents reported their child comparing themselves negatively to either their twin or to peers. Sometimes, this was something the child already did (and they were worried they would do it more); other times parents reported that such comparisons had started following the NCMP measurement and feedback, ‘*[Child Name] was very upset & began comparing herself to friends. Even now she focuses on her weight’* (P13412, ‘Overweight’). Although parents across different categories highlighted concern around potential negative impacts on children, only parents of children categorised as overweight (or who had stated their child was close to the boundary of ‘overweight’) voiced that they had directly observed negative impacts on their child.

The lack of support for children categorised as underweight was frequently noted by parents, who criticised the programme’s focus on overweight. Parents of children categorised as underweight highlighted a lack of support following the NCMP feedback, ‘*…but felt they should have made suggestions or recommendations’* (P10862, ‘Underweight’). Some parents used this space to explain that they disagreed with the NCMP itself or with weight measurement approaches generally, ‘*I think you have to balance what the scales are telling you along with the body shape and dress size to truly try identify if your child is unhealthily underweight or overweight…’* (P12361, ‘Overweight’). Some parents also highlighted taking different approaches at home, such as focussing on other aspects of the child and their lives, rather than their weight status. Some felt that the feedback provided no useful information or nothing they did not already know.

## 4. Discussion

The current study explored parents’ emotional and behavioural responses to feedback on their child(ren)’s weight status from the NCMP using a large population‐based sample. Parents were less likely to agree with the NCMP feedback if their child was not classed as ‘healthy weight.’ Between one‐third (for ‘underweight’) and one‐half (for ‘overweight’ or ‘very overweight’) of parents said they took action based on the feedback. For children categorised as underweight, the most common action was contacting a health professional, while parents of children categorised as overweight or obese most commonly encouraged their child(ren) to eat more healthily and be more active. Our thematic analysis cultivated themes of emotions, conclusions about child’s well‐being, justification, distrusting the assigned weight category and the programme being the problem.

Emotions elicited by the NCMP were dependent on the feedback received, with parents reacting positively if their child was categorised as ‘healthy weight,’ compared to expressing worry, anger or being upset if he or she was categorised as an alternative weight status. Conclusions were drawn about children’s well‐being based on the categorisation, again following the same positive and negative pattern. Some parents whose children were categorised as an alternative weight looked to take action. This is supportive of the secondary aim of the NCMP – to communicate children’s weight status to parents – but there remains a question around the utility of providing weight status feedback when parents do not feel adequately supported to take action. Not all LAs provide weight management services for children; a recent study showed that only 23% of NHS trusts offered weight management services for children and, where a service was provided, there was variability in eligibility criteria, funding source and intervention format [20]. In the context of children categorised as having underweight, parents in this study highlighted that there is even less support available.

In addition, many parents questioned or dismissed the weight category assigned to their child. Parents typically dismissed feedback based on other explanations not being considered, for instance, the child’s physical build, diet or activity level. This aligns with previous work highlighting that parents viewed the NCMP feedback as less credible because it does not consider the wider context such as the individual child’s lifestyle and other factors influencing their weight [12, 13, 21]. The use of BMI as a measurement of weight status in children has been criticised by researchers [22], especially in Reception Year children [15], as has using BMI without adjustment for ethnicity [23]. BMI is a measure of excess weight, rather than excess fat or fat distribution, and does not distinguish between fat‐ and fat‐free mass [24]. As such, using BMI and another measure of adiposity, such as waist circumference, may provide more accurate feedback [25]. The 2025 NICE guidelines also recommend using weight‐to‐stature ratio alongside percentile to classify weight status in children [26]. In addition, body fat tends to accumulate during adolescence, some of which is attributed to normal growth [27]. At 10–11 years, children will be at different stages of puberty and therefore experiencing different growth trajectories, which may influence weight changes around this age. Therefore, a snapshot can be unhelpful and potentially harmful if it contributes to comparison with others, low self‐confidence or the onset of dieting. Thus, tracking individual trajectories in BMI and waist circumference may be useful for assessing changes in adolescent adiposity, whereas a single BMI measurement can be inaccurate or unhelpful – and in some cases problematic – for individuals. However, we recognise that this is not feasible in the context of the NCMP as a population‐level programme.

In addition, many parents dismissed the feedback by pointing to their child’s diet, activity levels or physical appearance, which may reflect limited parent understanding of the broader factors that shape weight status — including gene–environment interactions, local environments and SEP. Because the NCMP presents BMI categories without this wider context, it does little to raise awareness of the complex causes of weight and may unintentionally reinforce blame or stigma. Providing greater contextual information could help parents make sense of the categorisation and reduce negative responses. At the same time, the NCMP remains valuable for tracking inequalities in weight status by SEP and geographical region.

Parents feared negative impacts of the NCMP on their own or other people’s children, with some reporting that they had observed their child comparing themselves negatively to others or suffering from low self‐esteem following participation in the NCMP. This supports previous work, where parents considered the potential risks of bullying, disordered eating and poor mental health as more important than future risks of being overweight [9]. Parents also previously reported concern about placing too much emphasis on weight, cultivating an unhealthy obsession with weight or giving their child a stigmatising label for the future and prompting possible weight‐related teasing [21, 28]. Some parents in the present study disagreed with the weight management approach, reporting taking a different approach at home. This aligns with previous work where parents cited health and happiness as more important than weight [13].

Our qualitative findings align with what we know about how child weight is viewed by parents [11–13], and how weight status is viewed in Western society [29]. Perceived parental responsibility for children’s weight status gives context to parents’ responses. Parents usually see providing a healthy lifestyle for their child to be their responsibility, and so their child’s weight status, by extension, is an indicator of how well they have been cared for. This may be reinforced by the fact that the NCMP feedback letter is directed to the parent. Similarly, reactions and responses suggest that parents view their child’s weight status as a reflection of whether or not they are fulfilling their parental responsibilities. This is demonstrated in the feelings of pride, happiness and relief typically articulated when a ‘healthy weight’ categorisation is received, and declarations of worry or upset at an alternative weight status. This is consistent with previous qualitative work within a smaller sample, which found that parents of children categorised as a ‘healthy weight’ often expressed pleasure and happiness, whereas those whose children were classified as overweight more commonly reported shock and self‐blame [12]. Explicit statements of parental responsibility, although few in our sample, were typically presented as inferred from either positive or negative feedback. A parent was either a ‘good’ parent fulfilling their responsibilities or a ‘bad’ parent depending on whether their child was categorised as ‘healthy weight’ or an alternative weight status, respectively [29]. These reactions reflect broader societal attitudes towards body weight that remain prevalent in our society. Parents’ responses often varied according to whether their child was classified as being of ‘healthy weight’ or another weight category, with negative emotions or justifications observed when a child was identified as overweight. Although much of this was inferred from the nature of parents’ responses, one parent explicitly stated a preference for their child to be classified as ‘underweight’ rather than ‘overweight.’

Our findings have implications relating to the NCMP feedback and its impact. The function of the NCMP feedback (most commonly in the form of a letter) is to communicate children’s weight status and, in cases where they are not categorised as a ‘healthy weight,’ to encourage positive lifestyle changes. However, our quantitative findings demonstrated that less than half the parents whose child received an alternative weight status acted on the feedback. On the other hand, many parents reported negative emotions relating to the feedback, and disagreement with the measurement, the NCMP generally or the weight measurement approach. This suggests that LAs do not always communicate weight status information to parents in a way that is useful for them. Engagement with parents around how best to communicate information about children’s weight, how to signpost support and what support pathways would be most helpful could be used to redevelop and standardise the feedback process. For example, co‐creation of NCMP communication or support packs for parents [30]. A similar initiative addressing how to talk to children about weight has been successfully implemented [31].

There is a clear lack of insight into how children experience the NCMP, especially 10‐ to–11‐year‐old (Year 6) children, who are likely to have more understanding of why they are being weighed but are also vulnerable to misunderstanding or bias from their peers. In particular, current research has not distinguished between experiences of the measurement itself (mandatory in all LAs) and experiences of receiving the feedback (via the optional feedback that the child may or may not see). Initial work from Nnyanzi [32] conducting semi‐structured interviews with children post‐measurement and feedback indicated that going through the NCMP caused annoyance, panic and worry among children who were classified with an alternative weight status, and ‘over‐sensitised’ all children to weight issues, regardless of their assigned weight category. There have also been concerns raised around unintended consequences of the NCMP measurement and feedback for children, such as bullying, weight stigma, disordered eating and poor mental health [9]. Epidemiological research has demonstrated a significant rise in reported weight loss attempts among children in England, including those categorised as ‘healthy weight,’ following the introduction of the NCMP letter [33]. Some of our parents reported (or alluded to) sharing the feedback with their children, with others describing changes in their children’s behaviour following the feedback, for example, comparing themselves to other children. Parents need support in deciding whether to share the feedback with their child and how; and signposting parents to existing resources around talking to children about weight and health is vital [31]. The information children receive around weight and shape at this stage in their development is vital as they progress into puberty and adolescence, especially given that the NCMP precedes the peak age of onset for eating disorders and mental health conditions [34, 35]. Future research should explore how children experience the NCMP, and implications of the NCMP on later quality of life [9], distinguishing between the impacts of being measured from the impacts of receiving weight‐related feedback.

A strength of the current study is the availability of qualitative data from a large contemporary sample of parents with children categorised across all weight statuses. In particular, this study included a sizeable proportion of children categorised as underweight (6.5%) compared to what is typically seen in qualitative research where sample sizes are often smaller or there is a focus on children categorised as overweight/obese. There is typically very little discussion about, or support provided for, this group in relation to the NCMP. Indeed, our findings highlighted parents’ criticisms of the lack of support offered for families with children categorised as being underweight, with some dismissing the NCMP approach because of the overemphasis on ‘overweight’. Our study provides important insights into the impact of the NCMP on parents of children classified as having underweight and highlights that it will be vital to include these parents in the co‐creation of any new NCMP communication materials. A major limitation of this study is that the majority of families in the Gemini cohort are of White ethnicity and a higher SEP. We might expect different results from different populations, e.g., in populations where food insecurity is higher, or cultural norms around body size ideal differ. In addition, the NCMP questions were optional within the survey, and only 55.8% of parents completed these questions. The responders were predominantly female and there may be gender differences in how mothers and fathers perceive or report in relation to their child’s weight and health. More research is needed to examine this, although it is worth noting that mothers typically take on the majority of responsibility when it comes to managing child healthcare [36]. It would also have been useful to ask parents about the impact of children’s height feedback, as well as their weight status (e.g. boys of a shorter stature).

An additional limitation is the time elapsed between children being part of the NCMP (10–11 years old) and when their parents completed questions on their responses to it (12–13 years old). It is possible that parents incorrectly recalled their children’s weight status (although given the emotional impact of children receiving an alternative weight status, we speculate this is unlikely) or the actions taken. In addition, qualitative interviews with LAs delivering Tier 2 weight management services (12–24 weeks exercise and lifestyle programmes often provided by local councils in England; unpublished data) highlighted that parents’ responses and their openness to considering behaviour change or attending a weight management service altered over time, with immediate responses being denial or defence and responses a few months later being more focussed on change. This aligns with previous work highlighting a ‘journey’ of emotional responses in parents of children classified as an alternative weight status [12]. A future study should look to seek responses to our NCMP questions immediately following receipt of the NCMP letter and a few months following the NCMP to discern changes in emotional responses over time, as well as whether behaviour changes were maintained.

## 5. Conclusions

The negative reactions of many parents whose child is classified as an alternative weight status to ‘healthy weight’ highlight the need to involve parents in shaping how information on children’s weight is communicated to parents, how support is signposted and what pathways should be implemented to increase parental uptake.

**Nomenclature**
UK: United Kingdom
NCMP: National Childhood Measurement Programme
SEP: Socioeconomic position

## Ethics Statement

Ethical approval has been granted by the University College London (UCL) Committee for the Ethics of non‐National Health, originally in 2007 and again for the continuation of the study in 2018 (Project ID: 1624/004). Written informed consent was provided by all Gemini families, and all aspects of data collection and storage followed the standards specified by this body.

## Disclosure

This original research has not previously appeared in a published Abstract or been posted on a preprint server. The views expressed are those of the authors and not necessarily those of the NIHR or the Department of Health and Social Care.

## Conflicts of Interest

C.L. is Co‐Director of the UK National Institute for Health and Care Research (NIHR) Policy Research Unit in Healthy Weight, which is undertaking research into children’s experiences of the NCMP. C.L. is also Co‐PI on an NIHR Policy Research Programme grant examining the impact of the NCMP measurement on children’s mental well‐being. The other authors declare no conflicts of interest.

## Author Contributions

F.S. conceptualised the study, wrote the analysis plan, conducted the qualitative and quantitative analysis and wrote the initial manuscript for publication. C.L., A.F. and A.K. developed the questionnaire and lead data collection. A.K. curated the dataset from Gemini and was a second coder of a section of the qualitative data. Z.N. conducted some of the quantitative analyses and was a second coder of a section of the qualitative data. F.S. cultivated initial themes, and A.K., Z.N., A.F. and C.L. supported the finalising of these themes and the interpretation of the quantitative results. C.L. secured funding for Gemini and provided access to the dataset. All authors contributed to preparing the final manuscript for publication.

## Funding

No funding was received for this research. C.L. is supported by funding from the National Institute for Health and Care Research (NIHR) Policy Research Unit in Healthy Weight (PR‐PRU‐0916‐21001; grant number 174868) and an NIHR Policy Research Programme grant (NIHR207678).

## Supporting Information

Additional supporting information can be found online in the Supporting Information section.

## Supporting information


**Supporting Information 1** Supporting Information File 1: SRQR Checklist – Completed Standards for Report Qualitative Research (SRQR) checklist detailing where the qualitative aspects of this work are reported within the manuscript.

**Supporting Information 2** Supporting Information File 2: Positionality statements – Positionality statements written by FS, AK and ZN detailing their background, research interests and potential biases that they were cognisant of during analysis.

**Supporting Information 3** Supporting Information File 3: Supporting results – Supporting results tables (S1–4) with additional descriptive information.

## Data Availability

The data and materials used in this study are not publicly available. Access to Gemini study data can be requested from the Gemini research team and will be granted where appropriate.
