# Supplementary material for: Parents’ Responses and Reactions to the National Childhood Measurement Programme in a Contemporary Sample of British Children: A Mixed‐Method Study
Source: J Obes. 2025 Dec 8;2025:1001038. doi: 10.1155/jobe/1001038 (PMC12767035; doi:10.1155/jobe/1001038)
Supplement: Supplementary file 2 — Supporting Information 2 Supporting Information File 2: Positionality statements – Positionality statements written by FS, AK and ZN detailing their background, research interests and potential biases that they were cognisant of during analysis. [file JOBE-2025-1001038-s002.pdf]

Researcher Reflections – exploring parents' responses to their twins' categorisation of weight status from the National Childhood Measurement Program (NCMP)

Author: Florence Sheen (Coder 1)

Reflection completed: Pre-familiarisation (14.06.23)

I am a researcher in the psychology of eating behaviour with a background in obesity and public health research. I am currently conducting a fellowship project exploring how children interpret messages around (healthy) eating and body weight, and how we can better communicate with children around eating and weight to be more inclusive and supportive. I have also recently completed a secondment with the Office for Health Improvement and Disparities (OHID) exploring Tier 2 weight management services in London (March 2023). Therefore, I have expertise around the psychology of obesity and how parents and children might feel about being part of a weight management service. I am cognisant of the stigma surrounding weight status, and the various messages a child might be exposed to about healthy eating and body weight that could be potentially unhelpful or destructive.

Although I have previously conducted qualitative research, specifically projects exploring how front-of-pack labels can inform parents decision-making around how to feed their infants and how inclusive and effective tier 2 weight management services in London are, I have not yet qualitatively explored how parents feel about their child's weight status. In addition, I do not have any children of my own. Although I have several friends who have young children, they are not usually part of my daily/everyday life. So, I do not experience, directly or indirectly, the day-to-day concerns, worries or problems that parents may encounter when trying to support their child(ren) to be healthy and happy. Therefore, I recognise that, although I have expertise and knowledge of obesity research, I am a novice with regards to how parents might feel and respond to being told that their child(ren) has been categorised as a particular weight status.

Given the controversy around the National Childhood Measurement Program (NCMP) as well as my own opinions around how devastating and unhelpful I believe it is to tell someone that they are living with overweight or obesity, I am expecting parent responses to receiving this information to be quite negative. When conducting my coding and generation of initial themes, I will be aware of my stance as looking at this from a research perspective, and with a somewhat negative view of the NCMP letter delivery. I will be mindful of keeping that separate from the unique experiences and perspectives that parents bring when asked how the feedback made them feel.

Author: Alice Kininmonth (Coder 2)

Reflection completed: Pre-coding (19.01.2024)

I am a research fellow in appetite research. My research investigates the role of eating behaviours, socioeconomic status and the environment on children's weight. I am currently undertaking research examining relationships between socioeconomic, environmental, psychological factors and family food environments and childhood obesity. I also work on a multidisciplinary project focussing on families with children during the first 1000 days. I have previously undertaking research into disordered eating and eating disorders and am aware of the negative impacts that weight stigma and weight shaming can have. I am a member of the Association for the study of obesity and am highly aware of the stigma faced by individuals living with overweight and obesity but also those with underweight.

I have previously conducted qualitative research as part of an evaluation of a Tier 3 weight management service. As part of this I interviewed adults living with obesity who had been through the tier 3 weight management service. I have never interviewed parents about their child's weight status nor interviewed children about their own weight. I do not have children of my own. I acknowledge that I do not understand first-hand how parents might feel and respond to being told that their child(ren) has been categorised as a particular weight status.

I am aware anecdotally of parents receiving the NCMP letter and being very upset and frustrated by the letter. I am conscious of this and will be cognisant of my preconception that the NCMP letter is unhelpful. I will be mindful of keep my preconception separate from the unique experiences and perspectives that parents bring when asked how the feedback made them feel.

Author: Zeynep Nas (Coder 3)

Reflection completed: Pre-coding (19.01.2024)

I am a postdoctoral research fellow in eating disorder research. My research focuses on the child and adolescent period, with a focus on the role of early life in eating behaviours and the development of eating disorders in adolescence and beyond. I have expertise in behaviour genetics research and currently undertake research relating to the genetic and environmental contributions to appetite and eating disorders. I am a member of the Association for the study of obesity and currently lead the London & South Eastern region network. I am highly aware of the stigma faced by individuals living with overweight and obesity but also those classed as underweight.

I have not previously conducted qualitative research but have personally taken part in a Tier 2 weight management service. I have never interviewed parents about their child's weight status nor interviewed children/adults about their own weight. I don't have children of my own. I have family and friends with younger children though I do not see them on a day-to-day basis. I therefore acknowledge that I do not understand first-hand how parents could feel and respond to being told that their child(ren) has been classed in a particular weight status category.

I am aware that parents receiving the NCMP letter can be very upset and frustrated by the outcome. I am conscious of this and will be mindful of my preconception that the NCMP letter can be unhelpful and worrisome for some. I will do my best to keep my preconception separate from the unique experiences and perspectives that parents bring when asked about the feedback and how it has made them feel.
