# Supplementary material for: Parents’ Responses and Reactions to the National Childhood Measurement Programme in a Contemporary Sample of British Children: A Mixed‐Method Study
Source: J Obes. 2025 Dec 8;2025:1001038. doi: 10.1155/jobe/1001038 (PMC12767035; doi:10.1155/jobe/1001038)
Supplement: Supplementary file 3 — Supporting Information 3 Supporting Information File 3: Supporting results – Supporting results tables (S1–4) with additional descriptive information. [file JOBE-2025-1001038-s003.docx]

**Supplementary Results**

*Twin-specific demographics*

*Table S1.* Combined sex of twin pairs

|  | Frequency | % |
| --- | --- | --- |
| Male | 197 | 34.7 |
| Female | 213 | 37.6 |
| Opposite sex | 157 | 27.7 |

Combined sex of twin pairs is based on baseline data, and so is present for all children (including where only 1 twin had NCMP data, n=15).

The majority of children were part of a dizygotic twin pair (n=373, 65.8%); 192 children were part of a monozygotic twin pair (33.9%). The zygosity of 2 twins was unknown (see Table S2).

*Table S2.* Combination of sex and zygosity of the children.

|  | Frequency | % |
| --- | --- | --- |
| Monozygotic male twins | 90 | 15.9 |
| Dizygotic male twins | 107 | 18.9 |
| Monozygotic female twins | 102 | 18.0 |
| Dizygotic female twins | 109 | 19.2 |
| Dizygotic twins of the opposite sex | 157 | 27.7 |
| Unknown | 2 | 0.4 |

Combination of sex and zygosity is based on repeat zygosity questionnaires and DNA.

*Table S3.* Congruency of weight status by zygosity (Values are Frequency (row %))

|  | Weight status not given^1^ | Congruent | Incongruent |
| --- | --- | --- | --- |
| MZ (n=192) | 14 (7.3) | 160 (83.3) | 18 (9.4) |
| DZ (n=373) | 27 (7.2) | 256 (68.6) | 90 (24.1) |
| Unknown (n=2) | 0 | 2 (100.0) | 0 |

^1^This includes response of ‘don’t remember’ present for one (n=1) or both (n=26) twins, or only one twin having having NCMP data (n=15).

*Table S4.* Parental agreement, whether they shared the NCMP feedback with their child, and action taken following the feedback split by assigned weight category (Values are n(row %))

|  | Parental Agreement | | | Shared with child | | | | Action taken | | | |  |
| --- | --- | --- | --- | --- | --- | --- | --- | --- | --- | --- | --- | --- |
|  | Yes | No^1^ | Prefer  not to say | | Yes | No | Don’t  remember | | Yes | No | Don’t  remember | |
| ‘Underweight’(n=37) | 29 (78.4) | 8 (21.6) | 0 (0) | | 26 (70.3) | 9 (24.3) | 2 (5.4) | | 11 (29.7) | 25 (67.6) | 1 (2.7) | |
| ‘Healthy weight’ (n=440) | 428 (97.3) | 10 (2.3) | 2 (0.5) | | 330 (75.0) | 58 (13.2) | 52 (11.8) | | 14 (3.2) | 423 (96.1) | 3 (0.7) | |
| ‘Overweight’(n=44) | 25 (56.8) | 18 (40.9) | 1 (2.3) | | 24 (54.5) | 16 (36.4) | 4 (9.1) | | 24 (54.5) | 18 (40.9) | 2 (4.5) | |
| ‘Very Overweight’ (n=20) | 10 (50.0) | 10 (50.0) | 0 (0) | | 12 (60.0) | 8 (40.0) | 0 (0) | | 9 (45.0) | 11 (55.0) | 0 (0) | |
| ‘Don’t remember’ (n=26) | 18 (69.2) | 4 (15.4) | 4 (15.4) | | 9 (34.6) | 0 (0) | 17 (65.4) | | 3 (11.5) | 21 (80.8) | 2 (7.7) | |

^1^ Note, it was not possible to determine what parents believed their child’s weight status had been at that time, because the only individuals who gave a value for ‘what did you think [st]’s weight status was at the time?’ were those who had not provided the NCMP measurement.
